# The molecular basis of endothelial cell plasticity

**DOI:** 10.1038/ncomms14361

**Published:** 2017-02-09

**Authors:** Elisabetta Dejana, Karen K. Hirschi, Michael Simons

**Affiliations:** 1 Vascular Biology Unit, FIRC Institute of Molecular Oncology, Milan 20129, Italy; 2 Department of Immunology, Genetics and Pathology, Uppsala University, Uppsala 751 85, Sweden; 3 Yale Cardiovasc. Res. Center, Departments of Internal Medicine, Genetics and Biomedical Engineering New Haven, Connecticut CT06511, USA; 4 Yale Cardiovascular Research Center, Department of Internal Medicine and Department of Cell Biology, Yale University School of Medicine, New Haven, Connecticut CT06511, USA

## Abstract

The endothelium is capable of remarkable plasticity. In the embryo, primitive endothelial cells differentiate to acquire arterial, venous or lymphatic fates. Certain endothelial cells also undergo hematopoietic transition giving rise to multi-lineage hematopoietic stem and progenitors while others acquire mesenchymal properties necessary for heart development. In the adult, maintenance of differentiated endothelial state is an active process requiring constant signalling input. The failure to do so leads to the development of endothelial-to-mesenchymal transition that plays an important role in pathogenesis of a number of diseases. A better understanding of these phenotypic changes may lead to development of new therapeutic interventions.

## Abstract (web summary)

Vascular endothelium possesses remarkable plasticity in response to cues from its surroundings, leading to great heterogeneity of endothelial cells in different vascular beds. Here the authors explain the molecular basis of endothelial plasticity during embryogenesis and in various diseases.

Endothelial cells, that form the early vascular plexus during embryo development, progress through a number of fate transitions until they achieve their highly differentiated adult state. At early stages of embryonic development, cells that will form the primitive vasculature exhibit a primordial, non-specialized endothelial phenotype. As the plexus is remodelled into specialized vascular structures, these primitive endothelial cells acquire specialized characteristics typical of arteries, veins or lymphatics. Subsequently, during organ vascularization, the endothelial cells differentiate further to adapt to the specific needs of the organ^1^.

In this review, we discuss key emerging concepts and challenges in the rapidly moving field of endothelial fate transition, including signalling pathways implicated in endothelial-to-hematopoietic cell transition (EHT) and endothelial-to-mesenchymal transition (EndMT), as well as physiological and pathological implications of these processes.

## Endothelial cell development and fate transitions during embryogenesis

The vasculature is among the first organ systems to develop during embryogenesis, and is essential for the growth, survival and function of all other organ systems. Blood vessels are composed of endothelial cells that form the inner, luminal layer and smooth muscle cells that form the surrounding vessel wall. During blood vessel development, endothelial cells are formed first, and undergo rapid expansion and coalescence into capillary plexi that are then remodeled into a circulatory network. Vascular remodelling and maturation involves coordinated migration, growth control and specification of arterial and venous endothelial subtypes, as well as smooth muscle cell recruitment.

As the vasculature is established within distinct organs, the endothelium therein is further phenotypically specialized to meet the needs of the tissue. For example, in the brain and retina, tight junctions are formed to create a barrier against infiltration of circulating factors and cells. In contrast, in tissues with filtration functions, such as the kidney and liver, the endothelium can be discontinuous and develop fenestrae to promote infiltration and extravasation of circulating factors.

Vascular endothelium also significantly contributes to the development of other organ systems, including blood and the heart. In these circumstances, endothelial cells undergo a fate transition into another cell type; that is, hematopoietic cells, or cardiac mesenchyme, respectively. The differentiation, specialization and fate transitions of endothelium during development are discussed herein.

### Endothelial cell differentiation

The emergence of primordial (non-specialized) endothelial cells is referred to as vasculogenesis and begins in the developing mammal shortly after gastrulation in the extraembryonic yolk sac. Endothelial cells are formed from mesodermal progenitors in response to signals from the adjacent visceral endoderm and coalescence into vascular plexi that are remodeled into circulatory networks during the process of angiogenesis.

Genetic manipulation studies in the mouse revealed that fibroblast growth factor 2 (FGF2 or bFGF) and bone morphogenetic protein 4 (BMP4) are not only critical for mesoderm formation, but also play an important role in endothelial cell differentiation.^2^ Indian hedgehog (IHH) signalling, likely mediated via BMP4 (ref. 3) also promotes endothelial cell development, and is sufficient to induce the formation of endothelial cells in mouse embryo explants that lack endoderm^2^. Vascular endothelial growth factor (VEGF-A) is another key regulator of vasculogenesis. It predominantly binds two receptors, VEGFR1 (Flt-1), which acts as a sink for bioactive VEGF-A, and VEGFR2 (Flk-1 or Kdr), which is required for vascular plexus development^4^. VEGFR2^^−/−^^ mouse embryonic stem cells generate endothelial cells, although they fail to propagate *in vitro*. Thus, VEGF-A may regulate the survival and/or propagation of endothelial cells, but not their fate specification.

Transcriptional regulators in the ETS family are known to play an important role in endothelial cell development, and the regulatory regions for almost all endothelial genes contain ETS binding sites^5^,^6^. ETS variant 2 (Etv2 or ER71/etsrp), in particular, regulates the differentiation of mesodermal progenitors toward an endothelial cell fate. It is restricted to VEGFR2-expressing mesodermal progenitors, and mice deficient in Etv2 lack a yolk sac vascular plexus, dorsal aortae and endocardium, despite normal mesoderm formation^7^. Overexpression of Etv2 *in vivo* leads to ectopic expression of endothelial-specific genes, suggesting it is necessary and sufficient for endothelial cell development^7^. FGF signalling is known to promote Ets-driven gene expression^8^, although we have much to learn about the coordination among signalling pathways and transcriptional regulators that mediate endothelial cell differentiation.

### Endothelial cell specialization

Once formed, primordial vasculature undergoes further differentiation and specialization, resulting in formation of distinct arterial, venous and lymphatic systems. Signalling pathways implicated in early endothelial cell development are also thought to play significant roles in arterial-venous specification. For example, during arterial-venous specification, VEGF-A binds to VEGFR2 and co-receptor neuropilin-1 (Nrp1), leading to activation of Notch signalling. Arterial-specific genes, including EphrinB2, are upregulated downstream of Notch signalling; whereas, venous-specific EphB4 expression is suppressed^9^. Inhibition of Notch signalling results in an arterial-to-venous fate switch^10^. Wnt signalling is also involved in the specification of arterial endothelial cells; β-catenin, a transcriptional co-activator of Wnt signalling pathway, upregulates Notch ligand Dll4 and promotes arterial specification^11^. In addition, Hedgehog acts upstream of VEGF-A via smoothened receptor to drive arterial endothelial cell specification and repress venous fate^12^,^13^. Venous endothelial cell specification is induced by chicken ovalbumin upstream promoter-transcription factor II (COUP-TFII). Endothelial-specific deletion of COUP-TFII leads to arterialization of veins, whereas ectopic expression results in fusion of veins and arteries^14^. Lymphatic endothelial cells are formed, in part, from a subset of endothelium within the cardinal vein; wherein, the co-expression of COUP-TF II and SOX18 leads to upregulation of PROX-1 that promotes lymphatic specification. The propagation of lymphatic endothelial cells is mediated by VEGFR3 signalling, driven by VEGF-C from the surrounding mesenchyme. Importantly, further stabilization and quiescence of the lymphatic system is mediated by FOXC2 and fluid shear stress, providing an essential link between biomechanical forces and endothelial cell identity^15^.

### Transition of endothelial cells to hematopoietic cells

As arterial and venous endothelial cells are being specified within the yolk sac, another type of endothelial cell is also developing. These are hemogenic endothelial cells that will give rise to multi-lineage hematopoietic progenitors through a process referred to as EHT. Hemogenic endothelial cells also form in other tissues throughout the course of gestation, including the placenta and umbilical vessels, as well as within the embryonic aorta-gonad-mesenephros (AGM) region where they give rise to hematopoietic stem cells (HSC)^16^. Other tissues observed to give rise to hematopoietic cells during development include the head vasculature^17^ and the endocardium^18^.

**Figure 1:**
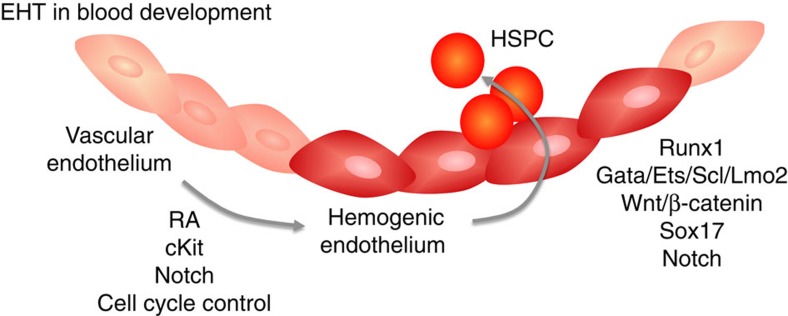
Schematic representation of endothelial-to-hematopoietic (EHT) transition and endothelial-to-mesenchymal (EndMT) transition during development. During definitive hematopoiesis, a subset of endothelial cells is specified to become hemogenic (dark red), and these cells give rise to hematopoietic stem and progenitor cells (HSPC) via EHT. The specification of hemogenic endothelial cells requires retinoic acid (RA) signalling, which leads to upregulation of c-Kit. Notch is activated downstream of c-Kit expression and controls endothelial cell cycle to enable hemogenic specification via mechanisms that are still unclear. The subsequent generation of HSPC requires transcription factor Runx1, and binding partners Gata, Ets, Scl and Lmo-2. Other factors involved in this process include Wnt/β-catenin, Sox17 and Notch, whose molecular roles and interactions are under study.

The process of blood production from the endothelium appears to require two distinct steps: hemogenic endothelial cell specification and EHT (depicted in Fig. 1). The initial specification of hemogenic endothelial cells involves some of the same signalling pathways implicated in arterial-venous specification, although it appears to be uniquely initiated by retinoic acid (RA) signalling. During vasculogenesis in the murine yolk sac, the first site of hemogenic endothelial cell specification, active RA is generated by retinaldehyde dehydrogenase 2 (Raldh2). Raldh2^−/−^ mutant embryos exhibit endothelial cell hyperproliferation, lack vascular remodeling and hemogenic endothelial cell development and die mid-gestation^19^. RA signalling is also critical for the development of hemogenic endothelial cells within the AGM^20^. In both tissues,∼90% of endothelial cells with active RA signalling exhibit a hemogenic phenotype, and ∼90% of hemogenic endothelial cells are undergoing active RA signalling^2^,^20^.

Signalling pathways that function downstream of RA in this process include c-Kit and Notch. c-Kit (CD117) is a receptor with tyrosine kinase activity that binds stem cell factor; its expression is a distinguishing feature of hemogenic endothelial cells, relative to non-blood forming endothelial cells^19^. c-Kit is known to be transcriptionally regulated by RA signalling, and re-expression of c-Kit in Raldh2^−/−^ mutants rescues hemogenic endothelial cell development and definitive hematopoiesis^21^.

Notch1 expression in endothelial cells is upregulated downstream of c-Kit^21^, and is essential for hemogenic endothelial specification, just as it is for arterial^22^ and lymphatic specification^23^. Notch signalling is involved in cell-fate decisions and cell differentiation, and embryos lacking Notch1, or Notch1 and Notch4, the only Notch receptors expressed by endothelial cells, exhibit abnormal vascular remodeling similar to Raldh2^−/−^ mutants^24^. Notch1, specifically, is expressed in the ventral wall of the dorsal aorta where hemogenic endothelium forms^25^ and the AGM of Notch1^−/−^ mutants exhibit decreased hematopoietic activity. Collectively, these data suggest that Notch signalling is important in hemogenic endothelial cell development; however, much needs to be learned about the role of Notch signalling in EHT. Some studies suggest Notch ligand distribution dictates the outcome of Notch signalling during definitive hematopoiesis in the AGM. That is, hemogenic specification is driven by low levels of Jag1-mediated Notch signalling. In the absence of Jag1, Dll4-mediated high Notch activity instead drives arterial specification^26^.

The formation of hematopoietic stem and progenitor cells (HSPC) from the endothelium has been most intensely studied in the AGM region. Therein, intra-aortic hematopoietic clusters are formed from hemogenic endothelial cells during EHT. The clusters are composed of pre-HSC that differentially express endothelial and hematopoietic markers including VE-cadherin c-Kit, Ly6a (Sca-1), CD41 and CD45, suggesting that a post-hemogenic endothelial cell intermediate may exist along the transition from endothelial cell to HSPC within these structures^27^,^28^,^29^. The dorsoventral polarity of the clusters emergence is thought to be guided by mesenchymally derived pro-hematopoietic ventralizing (VEGF, FGF2, TGFβ, BMP4) or anti-hematopoietic dorsalizing (EGF and TGFα) factors, which affect the expression of critical hematopoietic transcription factors involved in EHT (ref. 30).

One such essential transcriptional regulator of EHT is Runx1 (AML1), a member of a family of transcription factors called core binding factors^31^. Runx1 is thought to repress the endothelial program, while activating the hematopoietic program during the EHT process^27^,^29^. Deletion of Runx1 does not prevent hemogenic endothelial cell specification, but prevents the transition of these cells to CD41+CD45+ hematopoietic cells^32^. The transition from endothelial to hematopoietic phenotype is in part controlled by binding of multiprotein complexes containing GATA, Ets^33^, and SCL factors to Runx1 enhancers that promotes HSC emergence^34^, as well as the loss of expression of genes associated with arterial identity^35^, at least within the AGM.

There is further evidence that Notch and Wnt pathways interact to generate HSC in the zebrafish embryo and also are involved in driving hematopoietic development from embryonic stem cells^36^,^37^. Wnt/β-catenin activity is transiently required in the AGM for emergence and generation of long-term HSC, as well as production of hematopoietic cells in vitro from AGM endothelial precursors^37^. Downstream of Wnt signalling in the AGM, is expression of transcription factor Sox17. Conditional loss of Sox17 in endothelial cells in the AGM leads to increased production of hematopoietic cells^35^, suggesting that Sox17 modulates the fate of hemogenic endothelium by actively repressing the hematopoietic program. Other studies show that enforced expression of Sox17 in mouse embryonic stem cells leads to increased blood-forming endothelial cells that generate T lymphocytes, through a mechanism involving Notch signalling^38^. Thus, more work is needed to understand the roles and interactions among these regulators of EHT.

Other factors, including inflammatory^39^,^40^,^41^,^42^ and G-protein coupled receptor signalling^43^,^44^, purine signalling^45^ and chromatin remodeling^46^,^47^ are also involved in promoting EHT, and their coordination with known regulators of this process are under investigation.

### Endothelial-to-mesenchymal transition in development

In addition to vascular endothelial cells within hematopoietic tissues undergoing EHT and giving rise to hematopoietic stem and progenitor cells, the specialized endothelial cells that line the heart (endocardial cells) also undergo EndMT, and give rise to mesenchymal cells necessary for proper heart development. Although all cells in the heart arise from one or more epithelial-to-mesenchymal transition, EndMT, specifically, generates valve progenitor cells that give rise to the mitral and tricuspid valves. EndMT also contributes to endocardial cushion formation, as well as to generation of cardiac fibroblasts and smooth muscle cells, but not cardiac myocytes.

These developmental mechanisms may well be recapitulated in adult valve disease, in cardiac fibrosis, and in myocardial responses to ischemic injury (see below). Thus, understanding what regulates EndMT during embryogenesis may provide insights needed to treat postnatal pathologies. In addition, since some of the signalling pathways that regulate developmental EndMT (TGFβ, BMP, Notch, Wnt/β-catenin)^48^ also play a role in EHT (Fig. 1), as well as postnatal EndMT (discussed in detail in subsequent sections), comparing these regulatory pathways may reveal common targets for therapy.

## Maintenance of adult endothelial homeostasis

The enormous phenotypic plasticity exhibited by endothelial cells is a direct reflection of their exposure to the environment full of growth factors, cytokines, rich in oxygen and mechanical stresses. It is not surprising, therefore, that maintenance of endothelial normalcy is an active processes requiring constant energy expenditure and signalling input. While still poorly understood, recent studies shed light on several active endothelial ‘maintenance' pathways. These include regulation of expression of key proteins such as VEGFR2 and FGFR1, maintenance of endothelial barrier function, suppression of apoptosis and prevention of the fate drift.

Fibroblast growth factors (FGFs) play a particularly important role in control of endothelial homeostasis. Even a transient withdrawal of the FGF signalling input leads to a progressive loss of endothelial cell-cell contacts, increased permeability and, eventually, compromise of vascular integrity^8^,^49^,^50^ while a more prolonged withdrawal leads to endothelial apoptosis^51^ and vascular rarefication^52^ including the loss of *vasa vasorum*^53^. In part, these changes are due to increased VE-cadherin phosphorylation at Y658 (and thus the loss of VE-cadherin-p120-catenin association that impairs adherence junctions), and in part to a decline in VEGFR2 levels (thereby increasing endothelial apoptosis). The former is a consequence of a decrease in FGF-dependent expression of the phosphatase Shp2 (ref. 54) while the latter is due to the requirement for the FGF-driven Ets/FOXC2 VEGFR2 transcription^8^. Since both VE-cadherin phosphorylation and VEGFR2 turnover are rapidly occurring processes, withdrawal of a continuous FGF signalling input rapidly leads to the above-described consequences^55^.

Another key role played by the FGF signalling input is maintenance of the endothelial cell fate. FGFs achieve this by blocking activation of TGFβ signalling cascade that is central to the induction of EndMT transition both in blood^56^,^57^ and lymphatic endothelial cells^58^ that is discussed below. Endothelial homeostasis is also maintained by VE-cadherin that is the cell specific major organizer of endothelial cell-to-cell adherens junctions^59^,^60^. The expression and clustering of VE-cadherin at cell–cell junctions not only maintains endothelial cell-to-cell adhesion but, through the interaction with a complex network of intracellular partners, transduces intracellular signals that mediate contact inhibition of cell growth, cell polarity and lumen formation. Therefore, conditions that disrupt endothelial junctions not only induce increase in vascular permeability by opening intercellular gaps but also change the endothelial cell responses to their environment and to the surrounding cells. For instance, when VE-cadherin junction organization is dismantled, gene transcription of endothelial cells is strongly modified, the cells tend to grow in multiple layers, are unable to form a correct vascular lumen and establish adhesion contacts with the surrounding pericytes and smooth muscle cells^61^,^62^,^63^.

At the molecular level, VE-cadherin is linked through its cytoplasmic domain to p120 catenin, β-catenin and plakoglobin. Furthermore, it can interact and modulate signalling of several growth factor receptors to promote contact inhibition of cell growth. For instance, VE-cadherin expression and clustering inhibits VEGFR2 and FGFR1 signalling while increases TGFβR complex organization and signalling^64^,^65^,^66^. Some phosphatases (VE-PTP, DEP-1, PTPu, Csk and SHP2)^61^,^67^,^68^ and kinases (such as Src or FAK)^69^,^70^,^71^ may also associate with the VE-cadherin complex and modulate cell signalling. This complex signalling system is dynamic and continuously adapting to different external conditions (shear stress, growth stimulation, increase in permeability) to maintain endothelial integrity^72^.

## Definition and occurrence of EndMT in various pathologies

In the absence of active input endothelial cells may either die or undergo EndMT, a process with certain similarities with the better understood epithelial-to-mesenchymal transition. Just as occurs during normal development, during postnatal EndMT, endothelial cells acquire mesenchymal characteristics such as an elongated, fibroblastoid morphology, increased motility, cytoskeletal modifications and cell-to-cell junction rearrangement. However, in the adult, they further become proliferative, thrombogenic and deposit large amounts of extracellular matrix.

**Figure 2:**
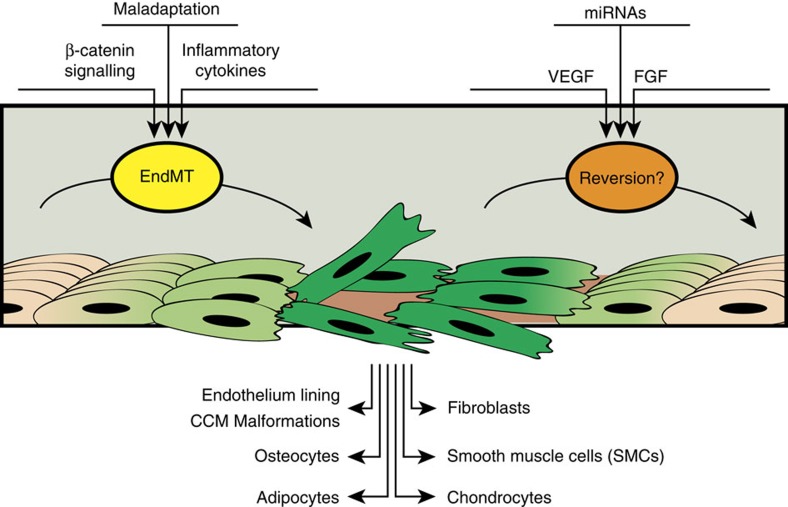
Schematic representation of endothelial-to-mesenchymal transition in the adult. In the adult, endothelial cells (flesh coloured) deprived of growth factors or exposed to inflammatory cytokines may undergo EndMT (see light and intense green cells representing the progression to EndMT) and acquire characteristics of fibroblasts, smooth muscle cells, osteocytes, adipocytes, chondrocytes or form vascular malformations such as CCM. Re-establishment of endothelial homeostasis by exposure to growth factors or to specific miRNAs may revert the mesenchymal phenotype (from intense green to flesh coloured cells).

**Figure 3:**
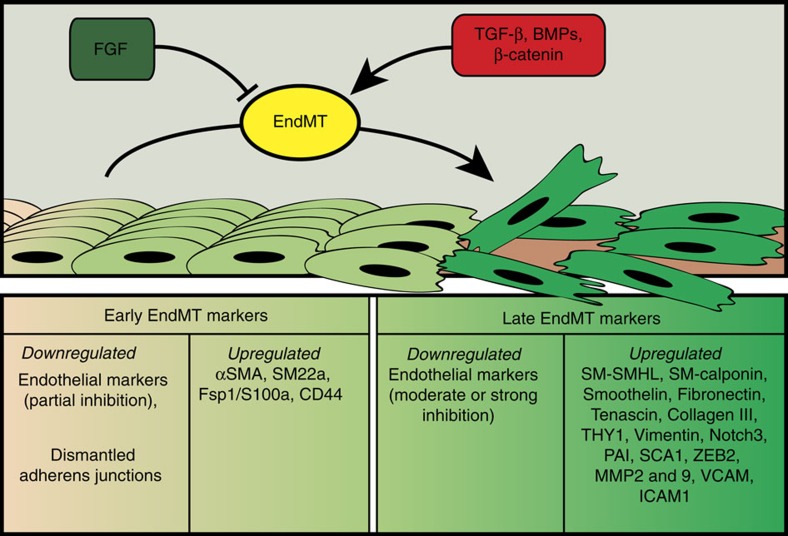
EndMT markers. Endothelial cells may undergo EndMT either from growth factor deprivation (FGF) or from activation of β-catenin, TGFβ, BMP pathways. EndMT progresses through different steps. The early endothelial response is characterized by a partial downregulation of endothelial markers, junction dismantling and up-regulation of some early mesenchymal markers. At later times, expression of endothelial markers further declines while more mesenchymal markers including matrix proteins, metallo-proteases or cytoskeletal proteins are up-regulated.

EndMT may lead to endothelial cells acquiring a variety of different mesenchymal fates (Fig. 2). As the result of this transformation, endothelial cells undergo a profound phenotypic change assuming the shape and properties of mesenchymal cells (fibroblasts, smooth muscle cells), including secretion of extracellular matrix proteins such as fibronectin and collagen, and expression of various leukocyte adhesion molecules. (see Fig. 3 reporting the list of specific markers)

At the cellular level, EndMT consequences include altered endothelial cell junction organization, loss of cell polarity, and increased cell proliferation and migratory capacity^73^. This results in a number of pathological consequences of considerable clinical significance in diseases ranging from cavernous cerebral malformations (CCM)^74^ to tissue fibrosis^75^,^76^,^77^, heterotopic ossification^78^,^79^, neointima formation^56^,^80^,^81^,^82^, atherosclerosis^81^ and cancer^83^,^84^.

The recognition of EndMT in tissues relies on detection of cells expressing both mesenchymal and endothelial markers. However, this approach assumes that cells that have undergone EndMT retain endothelial marker expression. While this is likely to be true immediately after the EndMT switch, at later stages endothelial cells may lose their endothelial markers (Fig. 3). Therefore, any immunocytochemical assessment of EndMT is an underestimate of the true frequency of this phenomenon. In addition, EndMT frequency varies depending on location and type of endothelial cells undergoing this process. Thus, in transplant settings, over 80% of luminal endothelial cells express mesenchymal markers while the frequency of this phenomenon elsewhere (for example, in the neointima) is much less^56^,^80^. Similarly, endothelial cells overlying the atherosclerotic plaque have a much higher EndMT incidence than nearby cells not in direct contact with the plaque^81^.

Fate-mapping has been employed to assess the true frequency of this phenomenon. For obvious reasons, however, this is possible in mice but not in clinical specimens. Fate-mapping involves activation of endothelial expression of a marker gene (typically LacZ) in adult mice using a tissue-specific Cre. Cdh5-CreER^T2^ is the most common driver line used for these studies^85^. It reaches high, but not 100% efficiency in most, but not all, endothelial beds so even this approach does not provide full assessment of EndMT frequency.

### EndMT in atherosclerosis and transplant arteriopathy

Recent studies documented EndMT contribution to vascular pathology both in transplant arteriosclerosis and atherosclerosis lesions. Both diseases are characterized by the growth of neointima that is composed of a mixture of smooth muscle cells, fibroblasts, mononuclear inflammatory and immune cells and extracellular matrix. In both cases chronic vascular inflammation, induced by certain immune mismatches in the case of transplant arteriopathy^86^ and lipid deposition and mechanical forces in the case of atherosclerosis^87^,^88^, are thought to play a central role in disease progression. EndMT has been detected in both conditions and likely plays a critical role in progression of both disease states.

Fate mapping using *Cdh5Cre;mTmG* fate mice in a mouse acute transplant rejection model showed that ∼10% of neointimal smooth muscle cells were of endothelial origin 2 weeks after transplant. At the same time, ∼80% of neointimal and ∼60% of luminal endothelial cells expressed mesenchymal markers indicating EndMT^56^,^80^. In agreement with these data, examination of patient samples from explanted rejected hearts found that ∼80% of coronary artery luminal endothelial and neointima cells were undergoing EndMT^56^,^80^.

Studies in other vascular injury models revealed a lower incidence of EndMT: 5% of neointimal SMC were of endothelial origin in the mouse wire injury model and 7% in vein-to-artery (inferior vena cava to aorta) graft model. Interestingly, in the latter case the incidence of EndMT increased from 3% at 2 weeks after grafting to 7% 4 weeks later, indicating that it is an ongoing process^56^,^80^. A study utilizing a somewhat different vein graft model (jugular vein to femoral artery) reported still higher incidence of EndMT: 28 to 50% at 5 weeks^82^. Similarly high incidence of EndMT has been observed in a mouse model using tissue-engineered vascular grafts. Here, EndMT frequency varied from 38 to 51% in occluded grafts (severe rejection) to 17% in patent grafts (less severe rejection)^89^. These variations in the observed EndMT extent likely relate to the severity of injury, the magnitude of the inflammatory response and differences in hemodynamic stress.

EndMT is equally prevalent in atherosclerosis, a progressive disease initiated by lipid deposition in parts of the arterial tree subjected to disturbed blood flow and is characterized by the gradual build-up of intraluminal plaques leading to reduction in distal tissue perfusion^88^. Some of the plaques are prone to rupture, an event than can lead to thrombosis and sudden death^90^. Chronic inflammation is thought to play a particularly important role in the disease progression although molecular details remain poorly understood^86^,^88^,^91^.

Examination of fate-mapped *Apoe*^−/−^ mice revealed that after four months of high fat diet ∼30% of luminal aortic endothelial cells were undergoing EndMT while no EndMT was detected in animals on the normal diet^81^. Similarly, examination of atherosclerotic plaques showed that about 35% of fibroblasts and “mesenchymal” cells were of endothelial origin^92^. Examination of human atherosclerotic vessels using immunocytochemical techniques, confirmed frequent occurrence of EndMT in coronary atherosclerosis^81^,^92^ and in carotid artery plaques^93^. Critically, there is a strong correlation (*r*=0.84) between the anatomically determined severity of coronary artery disease and the extent of EndMT in luminal endothelial cells^81^,^92^ in human coronary arteries.

One of the hallmarks of EndMT is activation of TGFβ signalling that, in turn, has been implicated in regulation of endothelial plasticity^92^,^94^. Indeed, in human coronary specimens there is a strong correlation between the extent of EndMT and the extent of activation of endothelial TGFβ signalling program^81^. The latter may directly contribute to atherosclerotic plaque growth as stimulation of EndMT increases atherosclerotic burden in Apoe^−/−^ mice^81^ and leads to formation of more unstable lesions characterized by larger necrotic cores and small fibrous caps^81^,^92^. However, the genetic/therapeutic proof of endothelial TGFβ signalling in atherosclerosis progression and regression is still absent.

### EndMT in cardiac fibrosis

Cardiac fibrosis, deposition of collagen and other extracellular matrix proteins in the myocardium play an important role in pathogenesis of a number of diseases from hypertension to coronary disease to various pressure- and volume-overload conditions. At the whole organ level, cardiac fibrosis leads to impaired chamber relaxation and may, eventually, result in reduction of cardiac output. Despite their obvious pathophysiologic importance, the origin of cardiac fibroblasts remains controversial in part because of the difficulty of accurately identifying cardiac fibroblasts using antibodies. Indeed, a number of “fibroblast” markers including fibroblast-specific protein 1 (FSP1), Thy1, vimentin and discoidin domain receptor family member 2 are expressed by other cell types including immune cells^95^. It is likely that a number of different populations are present that may not express a common antigen.

An early study that used Tie1Cre fate mapping suggested that EndMT may contribute significantly to cardiac fibrosis after myocardial infarction by increasing cardiac fibroblast population^75^. However, the employed approach did not distinguish between the expansion of a pre-existing Tie1+ population of fibroblasts vs. EndMT-derived expansion of the fibroblast pool. Indeed, a subsequent study demonstrated the existence of a Tie2-derived cardiac fibroblast population under normal conditions^96^.

However, the vast majority of fibrosis was accounted for by the pre-existing cardiac fibroblast pool and not by newly EndMT-derived fibroblast population^96^,^97^.

At the same time, ischemic injury induces the appearance of endothelium-like features in cardiac fibroblasts, the process referred to as mesenchymal to endothelial transition^98^. The fibroblast-derived endothelial cells exhibit functional and anatomical characteristics of normal endothelial cells including the ability to form vascular tubes and integrate into the newly forming vasculature. The process appears to be mediated by activation of p53-depedent gene transcription^98^ but many details remain poorly understood and unexplored.

### EndMT in CCM

CCM is a vascular disease that affects almost exclusively the venous microvasculature of the central nervous system and the retina^99^. CCM cavernomas are mulberry-like malformations that lack the support of mural cells and tend to bleed causing neurological problems such as headache, seizure and eventually hemorrhagic stroke. This pathology occurs either as sporadic or hereditary disease with an overall prevalence of up to 0.5% of the human population. The hereditary form is an autosomal dominant disease induced by the loss of function mutation of any of three genes named *Ccm* 1(Krit1), *Ccm* 2 (Osm) and *Ccm* 3 (Pdcd10)^100^. The only therapy available so far is neurosurgery that, however, may be dangerous depending on the location of the malformation.

Murine models have been created through endothelial specific inactivation of any one of the three CCM genes^74^,^101^,^102^. The morphology of the malformations as well as the specific cerebral localization are comparable to the human disease.

Either *Ccm1* or *Ccm3* null endothelial cells lining cavernomas lumens have dismantled cell–cell junctions and exhibit a typical EndMT phenotype switch^74^,^103^, including co-expression of endothelial and mesenchymal markers (Fig. 3). This switch in phenotype may explain many features of this disease, EndMT entails the loss of cell polarity and of contact inhibition of growth thus leading to formation of the typically enlarged and irregular lumen of the cavernomas. However, other mechanisms triggered by the absence of CCM genes may also contribute to the development of the malformations as discussed below in more detail.

### Other pathologies associated with EndMT

In the tumour stroma EndMT may be a source of cancer-associated fibroblasts and may contribute, in this way, to cancer progression. The key causative factor has not been defined yet but it is likely that TGFβ is involved. Cells undergoing EndMT in experimental tumors are particularly enriched at the invasive front of the tumor likely favouring invasion^83^,^84^. Furthermore, EndMT may play a positive role in metastatic cancer cell intravasation^104^. During this process the endothelium undergoing EndMT presents dismantled junctions and cytoskeletal contractility favoring the passage of tumour cells.

The actual contribution of EndMT to the overall number of fibroblasts in the tumour stroma, however, remains unclear. Taking into account the extreme functional variability of the different subsets of tumor associated fibroblasts, we do not know whether, and in which way, these endothelial-derived fibroblasts may differ in function from the other fibroblast populations present in the tumour stroma.

EndMT has also been proposed as the source of myofibroblasts in the kidney. Myofibroblasts are the dominant collagen-producing cells in many pathologies including organ fibrosis and may substantially contribute to kidney fibrosis and dysfunction^105^,^106^. In the mouse experimental kidney fibrosis model induced by unilateral ureteral obstruction, the total pool of myofibroblasts is composed of cells of different origins. Up to 50% originate from proliferation of local tissue-resident fibroblasts, while the EndMT transition gave origin to ∼10% of these cells. 35% of the nonproliferating myofibroblasts derive from bone marrow and the epithelial-to-mesenchymal transition contributes the remaining 5% (ref. 106).

Ablation of TGFβR2 in αSMA-positive cells significantly reduced the number of myofibroblasts recruited in the area of fibrosis underlining the importance of TGFβ signalling in this process. EndMT has also been implicated in the progression of other pathologies such as chronic obstructive pulmonary disease (COPD), pulmonary fibrosis, portal hypertension, heterotopic ossification systemic sclerosis, diabetic renal interstitial fibrosis and others. For space restriction we cannot discuss in detail each of them although many of the general characteristics of EndMT discussed above can apply also to these pathologies^77^,^94^,^107^,^108^

## Signalling pathways in EndMT

Given the extensive occurrence of EndMT, it naturally becomes important to understand the molecular signals and pathways leading to its development (Table 1). While there is a general agreement that endothelial TGFβ signalling is critical to postnatal EndMT induction, just as it is during development, the mode of its activation and contributory signalling pathways are subjects of intense research.

**Figure 4:**
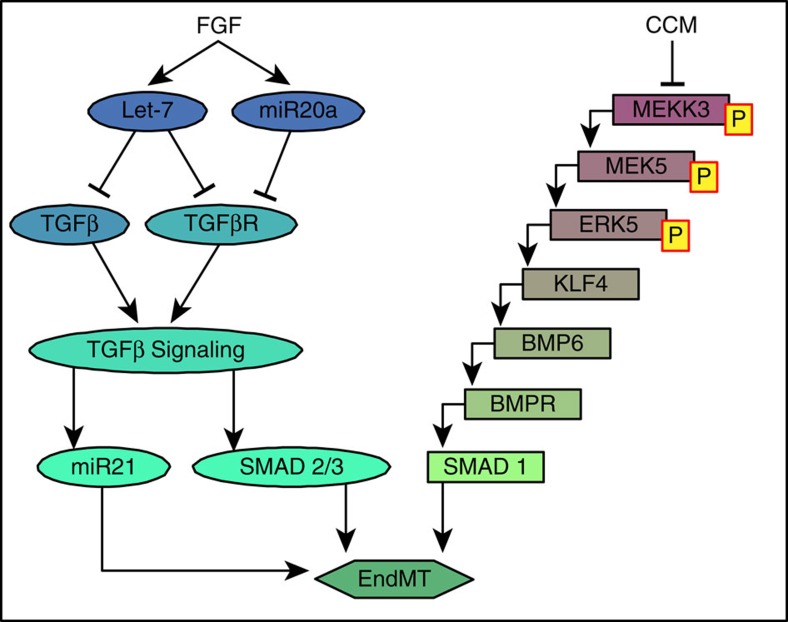
Signalling pathways inducing EndMT. Endothelial cells undergo EndMT as a result of the loss of FGF or CCM protective inputs. In the former case (left signalling sequence) reduction in let-7 levels results in increase expression of TGFβ family members' expression and activation of TGFβ signalling. MiR20a is induced by FGF and contributes to inhibition of EndMT by inhibiting TGFβ receptors signalling. Conversely, miR 21 is downstream TGFβ signalling and reduces EndMT development. In the latter (right signalling sequence), the loss of CCM inhibitory input activate MEKK3/MEK5/ERK5 cascade resulting in induction of BMP/Smad signalling.

A number of triggers have been proposed as EndMT drivers. The two best characterized are the activation of the MEK5/ERK5/BMP pathway after the loss of *Ccm*1 or *Ccm*2 gene expression^109^,^110^ and the activation of TGFβR signalling due to the loss of FGFR1 signalling input and decline in let-7 expression^56^,^80^,^81^ (Fig. 4) The former pathway is an important driver for EndMT in CCM settings while the latter is active in graft stenosis and atherosclerosis. Other proposed EndMT drivers include Wnt/β-catenin signalling that is known to act synergistically with TGFβ to promote EndMT in endocardial cells and in cavernomas of *Ccm*3 deficient mice^103^,^111^ and certain miRs such as: miR-20a that is down stream of FGF2 and targets TGFβR1 and R2 inhibiting EndMT^57^ and miR-21 that is induced by TGFβ signalling and promotes EndMT. Antagomir against miR-21 *in vivo* exerts antifibrotic effects by blocking End MT^112^.

*Ccm*1 ablation leads to the activation of a MEKK3-MEK5-ERK5-MEF2 signalling axis that induces a strong increase in Kruppel-like factor 4 (Klf4) in ECs *in vivo.* Klf4 transcriptional activity is responsible for the EndMT occurring in *Ccm*1-null endothelial cells. This transcription factor acts both by directly upregulating EndMT markers such as Fsp1, Sca 1, ID1 and also by increasing BMP6 and TGFβ signalling. In endothelial-specific *Ccm*1 and *Klf4* double knockout mice, the cerebral cavernomas were strongly reduced and mortality essentially abrogated^109^.

Another study confirmed the role of KLF2/4 transcription factors in the development of CCM but did not observe β-catenin or Smad signalling in endothelial cells of *Ccm -*deficient vessels^113^. These differences may be explained by the use of a different experimental model. The *Ccm*1 and *Ccm*2 KO models used by Zhou *et al*.^113^ exhibit a slower progression of the disease than the *Ccm1* and *3* knockout models used in previous studies^74^,^103^,^109^.

Studies of human tissues demonstrated that not only both Klf4 and pSmad 3 were upregulated in the endothelium lining human familial and sporadic CCM malformations, but that this was accompanied by a substantial upregulation of EndMT markers such as S100a4, αSMA, fibronectin, N-cadherin and ID1. Overall, these data confirm the importance of Smad signalling and EndMT switch in the endothelium lining CCM malformations in human patients^109^,^114^.

Other pathways may also contribute to the development and evolution of CCM including Notch, β1 integrins, autophagy, oxygen radicals, angiopoietin 2 and others^101^,^110^,^115^,^116^,^117^. However, their role in the induction of EndMT has not been investigated yet.

A series of studies have established the important role played by FGF signalling in control of the TGFβ pathway. FGF signalling is activated by an FGF binding to a cognate FGF receptor (FGFR); this is followed by phosphorylation of an adaptor molecule FRS2α that serves as a docking site for Shp2 and Grb2 that in turn act as scaffold for a series of intracellular molecules, eventually culminating in activation of MAPK and AKT^118^,^119^.Of the high affinity FGF receptor tyrosine kinases, FGFR1 has the highest level of expression in the endothelium^80^. Endothelial-specific deletion of FGFR1 alone or together with FGFR2 (expressed at very low levels) has no effect on vascular development,but impairs vasculature response to injury^120^ and sets the stage for EndMT induction^121^.

One of the consequences of endothelial-specific FRS2α or FGFR1 deletion is a dramatic (a 20- to 120-fold) reduction in expression of let-7 miRNA family (Fig. 4). Mouse and human let-7 miRNAs are the orthologs of *C.elegans* let-7 miRNA that plays an important role in development by controlling cell differentiation and proliferation^122^. Among let-7 targets is TGFβR1, and a decline in these miRNAs' expression leads to a large increase in the receptor's levels, along with that of other TGFβ family genes, including a 40-fold increase in TGFβ2 levels. This increase in both TGFβ ligands and receptors expression leads to activation of TGFβ signalling and development of EndMT^56^,^80^. Importantly, restoration of endothelial let-7 expression suppresses EndMT development *in vitro* and *in vivo*. The let-7/TGFβ link extends past the endothelium: suppression of its expression by Lin-28, a known negative regulator of let-7 biogenesis^122^, upregulates TGFβ-driven collagen production in glomerular mesangial cells^123^.

These data raise the question of the pathological importance of the FGFR-let-7-TGFβR pathway. Recent studies have shown that endothelial FGFR1 expression is repressed by the presence of certain inflammatory cytokines, including TNFα, IL1β and interferon-γ. Therefore, a decline in FGR1 levels may be anticipated in the setting of chronic vascular inflammation. Indeed, vascular inflammation is a hallmark of both transplant arteriopathy and atherosclerosis and in both settings there is a pronounced decrease in FGFR1 levels.

Abnormal shear stress may be another EndMT-inducing stimulus, both in development and in adult settings^124^. EndMT induction during development is critical to proper cardiac morphogenesis: areas of high shear stress such as the atrioventricular canal and cardiac outflow tract induction of EndMT, due to activation of TGFβ signalling^125^ and, indeed, EndMT is necessary for endocardial cushions morphogenesis^126^.

Studies using cultured umbilical vein endothelial cells demonstrated that uniform linear shear stress suppresses EndMT while inducing expression of Klf2 and Klf4 transcription factors. The latter represses arterial inflammation by binding to nuclear factor-kappa-B^127^. This was mediated by the activation of MEK5/ERK5 signalling as ERK5 silencing resulted in EndMT induction^93^,^125^. However, these same transcription factors may induce EndMT^109^ and cause major problems in heart and vascular development in *Ccm-*deficient animal models^110^,^128^.

The finding of important roles played by Klf4 and Klf2 in CCM is certainly intriguing. Klf4 is a zinc finger protein that also functions in pluripotent stem cell induction but it is also a regulator of endothelial activation in response to pro-inflammatory stimuli and TGFβ signalling^127^,^129^. In CCM lesions, both Klf4 and Klf2 are strongly upregulated but both transcription factors are also detectable in normal arteries and veins in resting conditions. This suggests that the level of Klf4 and Klf2 expression may dictate different types of cellular responses. Similarly to the strong induction of its expression seen in CCM lesions, Klf4 is also upregulated following the loss of endothelial FGF signalling input^80^, another EndMT trigger, although its role in this form of EndMT has not been established.

FGF signalling may also be involved in the shear stress-EndMT link. Atherosclerosis-prone areas of the arterial vasculature subjected to high shear stress demonstrate a reduction in FGFR1 expression and the appearance of low-level EndMT markers expression while atherosclerosis-resistant areas exhibit high FGFR1 expression^81^. It is interesting to speculate that a combination of vessel wall inflammation and abnormal shear stress may account for the development of EndMT in a variety of vascular diseases. Thus, EndMT may represent the key mechanism linking abnormal mechanical stress and inflammation-dependent disease progression.

Among miRNAs, miR-20a has been shown to be reduced during EndMT and restoration of its expression correlated with EndMT regression^57^. Under normal conditions, FGF signalling maintains miR-20a expression and decreased EC FGF signalling, as observed in atherosclerosis, would be expected to lead to the reduction in miR-20a expression^57^. These predictions are in agreement with the data implicating decreased FGF signalling in the initiation of EndMT and atherosclerosis progression^56^,^80^,^81^. In contrast, miR-21 is expressed downstream of TGFβ and activation of TGFβ signalling leads to its overexpression and the appearance of EndMT. Importantly, blockade of endothelial miR-21 expression reduced EndMT^112^. These two miRNAs, thus, act respectively upstream (miR-20a) and downstream (miR-21) of activated TGFβ signalling (Fig. 4).

## Concluding remarks

Whilst the newly formed endothelial cells are in a primordial, non-specialized state, they possess tremendous potential to specialize their phenotype to perform a range of functions, as well as give rise to other cell types necessary for blood and heart development. Thus, understanding the molecular mechanisms that govern these phenotypic changes and fate transitions, including EHT and EndMT, will be key to developing therapies to treat adult pathologies during which these processes have gone awry.

After birth, EndMT may have a pathological significance in different diseases and it is unclear whether it may be a reaction to injury or ‘maladaptation'. One of the consequences of EndMT is the increase in mesenchymal cells (fibroblasts, pericytes and smooth muscle cells and so on), and deposition of the extracellular matrix that increases fibrosis in case of inflammation. However, the percentage of endothelial-derived fibroblasts or smooth muscle cells may be limited and not substantial enough to significantly contribute to fibrosis and scar formation.

In CCM, EndMT-derived endothelial cells show dismantled junctions, VE- to N-cadherin switch, loss of polarity and hyperproliferation. These pathological reactions lead to vascular malformations and vessel fragility. In atherosclerosis, EndMT contributes to plaque formation, a process that leads to arterial lumen narrowing, by increasing the number of mesenchymal cells, but also by producing the extracellular matrix proteins and growth factors that indirectly increase mesenchymal proliferation. Furthermore, luminal endothelial cells that have undergone EndMT, express high levels of leukocyte binding molecules thereby promoting their influx into the vessel wall.

This sets up a positive-feedback loop that likely plays a key role in the relentless progression of atherosclerotic cardiovascular disease. EndMT may, therefore, contribute in a variety of ways to the progression of several pathologies. Increasing our knowledge of this process may help to design more specific pharmacological inhibitors able to interfere with endothelial fate transitions at the right time and in the right location.

## Additional information

**How to cite this article:** Dejana, E. *et al*. The molecular basis of endothelial cell plasticity. *Nat. Commun.* **8,** 14361 doi: 10.1038/ncomms14361 (2017).

**Publisher's note:** Springer Nature remains neutral with regard to jurisdictional claims in published maps and institutional affiliations.

## Figures and Tables

**Table 1:** Time course of EndMT signalling pathways in different experimental *in vivo* models.

**Mouse model**	**Pathway**	**Upregulated within**	**Downregulated within**	**Reference**
Mouse arteriosclerosis model	SMAD 2/3	2 weeks		80
Mouse vein graft adaptation model	SMAD 2/3	4 weeks		80
Human-to-mouse arterial transplant model	SMAD 2/3	4 weeks		80
Atherosclerosis model	SMAD 2/3	4 months		81
Mouse vein graft adaptation model	SMAD 2/3	3 and 7 days	at 14 days, undetected at 35 days	82
Mouse vein graft adaptation model	SMAD 1/5/8	3 days	no decline up to 35 days	82
Mouse vein graft adaptation model	Slug and Twist	3–14 days	at 35 days	82
Mouse vein graft adaptation model	Snail	0–7 days	no decline up to 35 days	82
CCM brain	SMAD1	9 dpn	no decline	103
CCM brain	Canonical Wnt signalling (β catenin)	3 dpn	9dpn	103,130
CCM brain	Klf4/Klf2	3 dpn	no decline	109

*dpn, days post natal.
